# Approaches and geographical locations of respectful maternity care research: A scoping review

**DOI:** 10.1371/journal.pone.0290434

**Published:** 2023-08-24

**Authors:** Hannah L. Shuman, Annika M. Grupp, Lauren A. Robb, Katherine G. Akers, Gurbani Bedi, Miloni A. Shah, Andrea Janis, Caroline G. Caldart, Urvashi Gupta, Janki K. Vaghasia, Aishwarya Panneerselvam, Aisha O. Kazeem, Ndidiamaka N. Amutah-Onukagha, Diane L. Levine

**Affiliations:** 1 Department of Internal Medicine, Wayne State University School of Medicine, Detroit, Michigan, United States of America; 2 Department of Public Health and Community Medicine, Tufts University, Boston, Massachusetts, United States of America; MUHAS: Muhimbili University of Health and Allied Sciences, UNITED REPUBLIC OF TANZANIA

## Abstract

**Background:**

Peripartum mistreatment of women contributes to maternal mortality across the globe and disproportionately affects vulnerable populations. While traditionally recognized in low/low-middle-income countries, the extent of research on respectful maternity care and the types of mistreatment occurring in high-income countries is not well understood. We conducted a scoping review to 1) map existing respectful maternity care research by location, country income level, and approach, 2) determine if high-income countries have been studied equally when compared to low/low-middle-income countries, and 3) analyze the types of disrespectful care found in high-income countries.

**Methods:**

A systematic search for published literature up to April 2021 using PubMed/MEDLINE, EMBASE, CINAHL Complete, and the Maternity & Infant Care Database was performed. Studies were included if they were full-length journal articles, published in any language, reporting original data on disrespectful maternal care received from healthcare providers during childbirth. Study location, country income level, types of mistreatment reported, and treatment interventions were extracted. This study was registered on PROSPERO, number CRD42021255337.

**Results:**

A total of 346 included studies were categorized by research approach, including direct labor observation, surveys, interviews, and focus groups. Interviews and surveys were the most common research approaches utilized (47% and 29% of all articles, respectively). Only 61 (17.6%) of these studies were conducted in high-income countries. The most common forms of mistreatment reported in high-income countries were lack of informed consent, emotional mistreatment, and stigma/discrimination.

**Conclusions:**

Mapping existing research on respectful maternity care by location and country income level reveals limited research in high-income countries and identifies a need for a more global approach. Furthermore, studies of respectful maternity care in high-income countries identify the occurrence of all forms of mistreatment, clashing with biases that suggest respectful maternity care is only an issue in low-income countries and calling for additional research to identify interventions that embrace an equitable, patient-centric empowerment model of maternity care.

## Introduction

From 2000 to 2017, the World Health Organization (WHO) estimated that the global maternal mortality ratio (MMR) saw a 38% reduction from 342 to 211 maternal deaths per 100,000 live births [1]. Access to healthcare facilities, education regarding safe sex practices, access to contraception, and other strategies have played crucial roles in the reduction in MMR during this time frame; however, global researchers and leaders still recognize the importance of continuing efforts to improve maternal care. The United Nation’s Sustainable Development Goals implemented in 2015 focus on reducing the global MMR to fewer than 70 per 100,000 live births by 2030 [2]. Recent research shows that disrespectful maternity care contributes to a higher MMR by deterring mothers from seeking care in hospital settings, which adversely affects mental health and contributes to poor health outcomes [3–12]. Therefore, emphasizing respectful maternity care (RMC) that empowers and promotes the autonomy of all people giving birth could be a powerful avenue for improving maternal wellbeing [2].

Over the past two decades, research on RMC globally has revealed persistent disrespectful care within clinical settings. Disrespectful care can range from physical and verbal abuse to poor pain management, lack of patient privacy, or patient discomfort—care factors that may not be thought of as inherently disrespectful. While disrespectful care may traditionally be thought to only impact low/low-middle-income countries (L/LMICs), the prevalence of disrespectful care during childbirth is not confined to L/LMICs. For example, in 2019, Vedam and colleagues’ Giving Voices to Mothers nationwide survey in the United States found that 28.1% of women giving birth in a hospital reported at least one type of mistreatment [13]. Another study in 2016 found that the MMR in the United States increased from 2000 to 2014—opposite to the direction of the global MMR [14].

In 2018, Bohren and colleagues attempted to standardize the measurement of mistreatment during childbirth by publishing and recommending the use of labor observation and survey tools by the research community [15, 16]. While Bohren and colleagues recommend labor observations and surveys as the most reliable way to measure mistreatment during childbirth, global studies have utilized a myriad of research approaches, including focus groups, surveys, social media searches, and interviews with patients or healthcare providers. Although the existence of disrespectful care in HICs may be recognized, it is currently unknown how much RMC research has taken place in HICs when compared to L/LMICs. Of the RMC research that exists, it is unknown whether the varied methodological approaches identify the same types of mistreatment in HICs. This scoping review aims to 1) map existing respectful maternity care research by location, country income level, and approach, 2) determine if HICs have been studied equally when compared to L/LMICs, and 3) analyze the types of disrespectful care found in HICs.

## Methods

### Search strategy

Literature searches were conducted in PubMed/MEDLINE (1809 to April 22, 2021), EMBASE (1937 to April 22, 2021), CINAHL Complete (1947 to April 22, 2021), and the Maternity & Infant Care Database (1971 to April 22, 2021). Based on a previously published search strategy by Bohren and colleagues [17], we combined search terms related to the concepts of perinatal care and disrespectful treatment to identify relevant studies (S2 File). Keywords and their associated subject headings were used as appropriate for each database. The reference lists of included studies were hand-searched to locate additional articles not captured by our database search. The search strategy was designed and implemented by a medical librarian.

### Article screening

**Fig 1:**
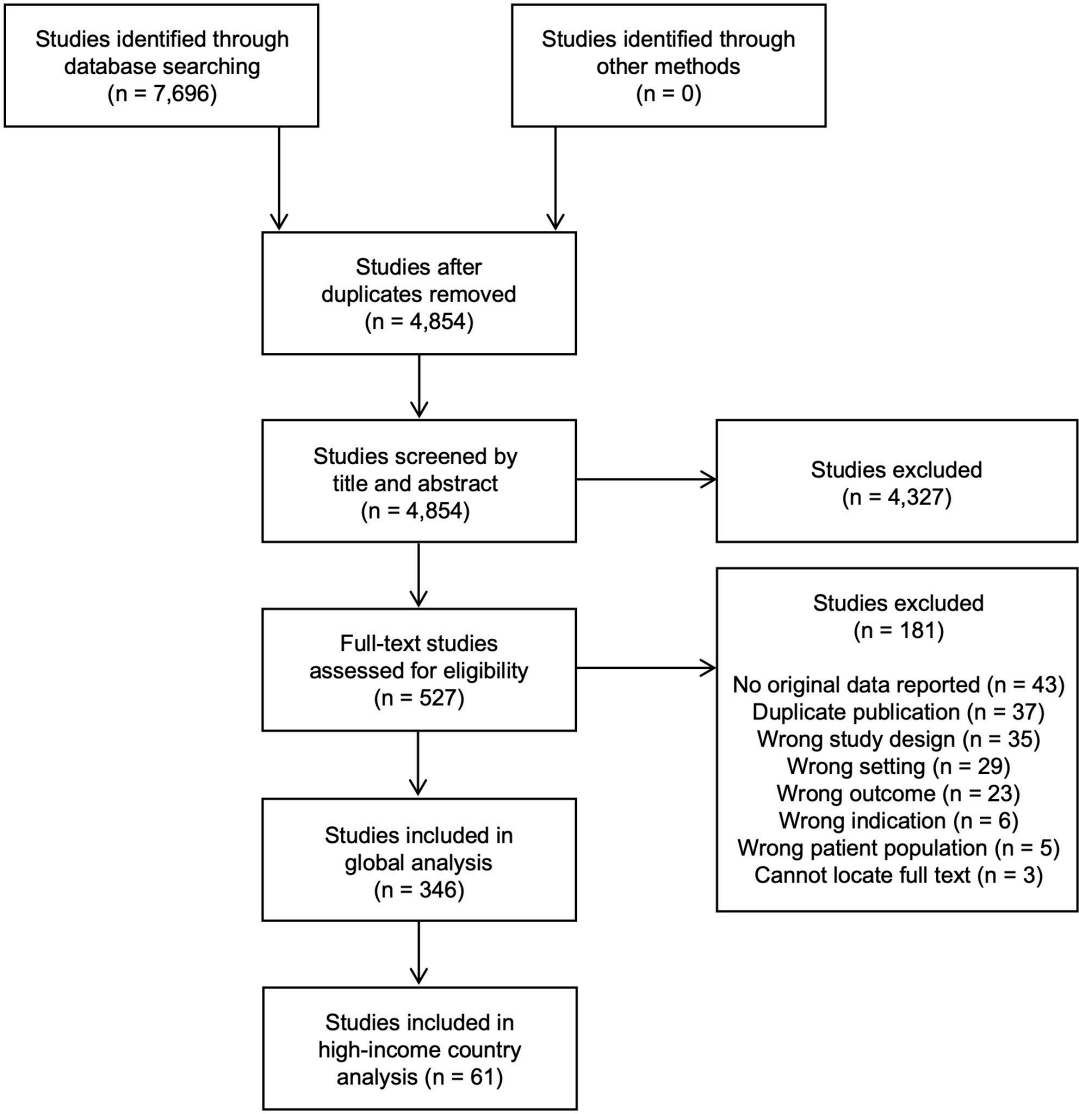
Flow diagram depicting search, article inclusion, and article exclusion process.

After de-duplicating the search results, studies underwent two rounds of screening based on their 1) title and abstract and 2) full text. In each round, studies were independently screened by two reviewers, and conflicts were resolved through discussion and consensus. Both rounds of screening were conducted using Covidence systematic review software (Veritas Health Innovation, Melbourne, Australia). Studies were included if they were full-length journal articles, published in any language, reporting original data on disrespectful maternal care received from healthcare providers during childbirth. Conference abstracts, editorials or opinion articles, and literature reviews were excluded (Fig 1).

### Data extraction

Data were extracted from included studies by two independent reviewers, and inconsistencies were resolved through discussion and consensus. Key characteristics of each study including publication date, location, and income group of the country were noted. We extracted information including study objectives, research approach(es), methods, results, and conclusions for each study (S1 Table). We also extracted data on specific types of mistreatment that were reported, who performed the mistreatment, and whether interventions were implemented (S2 Table). Types of mistreatment were developed from Bohren and colleague’s typology for categorizing mistreatment during childbirth [17]. Types of disrespectful care that fell into multiple categories were included in all categories that fit. For example, certain types of verbal mistreatment can also be considered emotional mistreatment and therefore were noted in both categories. Studies were then categorized by research approach for further analysis. For studies that employed multiple research approaches, the study was categorized and listed accordingly for each approach. This study was registered on PROSPERO, number CRD42021255337.

## Results

### Overview

Our literature search retrieved 7,696 studies. After removal of duplicates, we screened 4,854 titles and abstracts and 527 full-text articles against our inclusion and exclusion criteria, resulting in the inclusion of 346 studies (S3 Table) (Fig 1).

**Fig 2:**
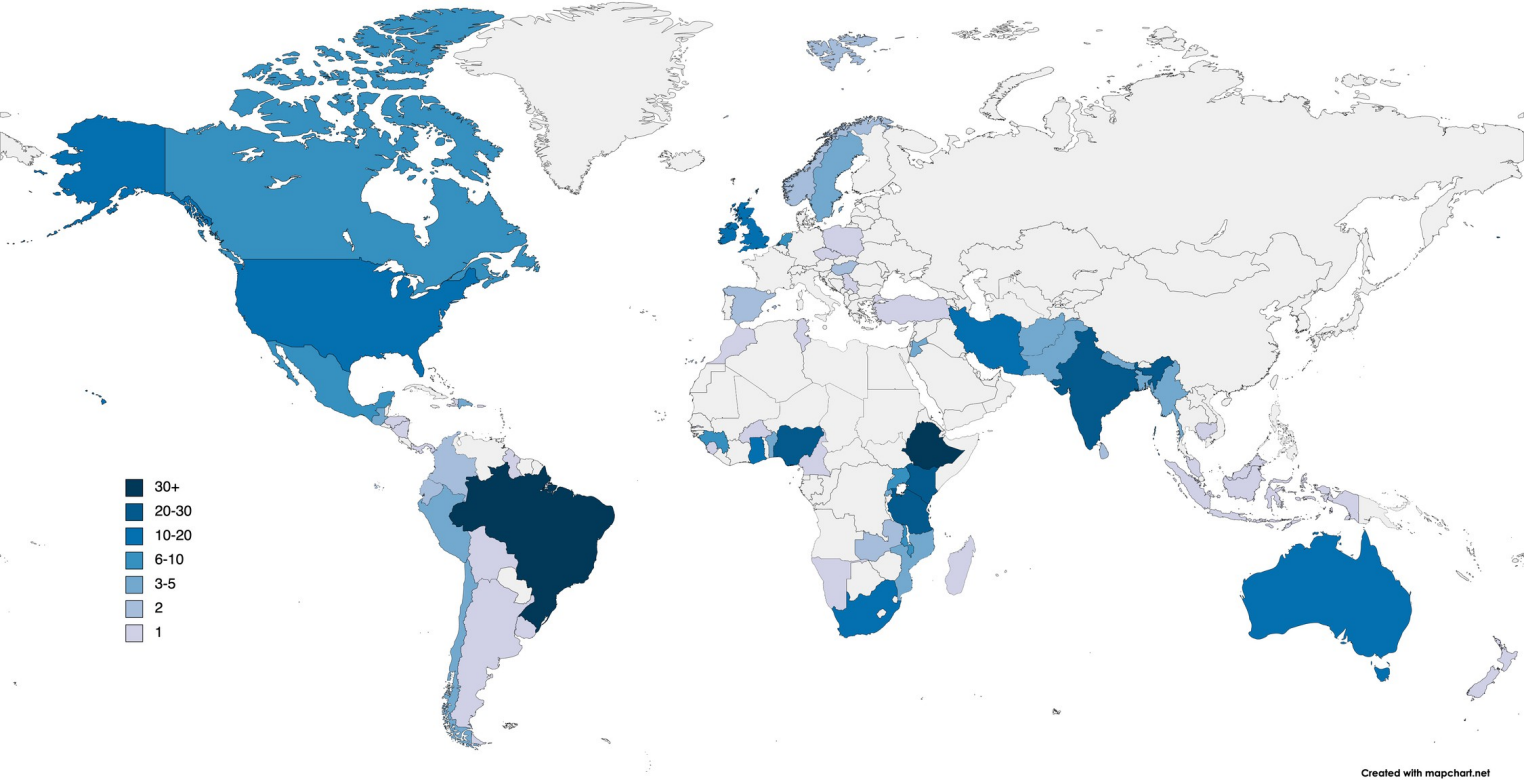
Global distribution of included studies by number of studies per country. Reprinted from Mapchart.net under a CC BY license, with permission from Mapchart, original copyright 2022.

To examine the distribution of RMC research globally, studies were organized by country (Fig 2) and country income category as determined by the World Bank [18] (Table 1). Among the 346 studies, 66 countries were represented, with Brazil, Ethiopia, India, and Tanzania being particularly prominent.

**Table 1:** Country income categories of included studies as determined by the World Bank.

High-income countries (HICs) (15 countries)	Upper-middle-income countries (UMICs) (16 countries)	Lower-middle-income countries (LMICs) (24 countries)	Low-income countries (LICs) (11 countries)
• Australia	• Argentina	• Bangladesh	• Afghanistan
• Canada	• Brazil	• Benin	• Burkina Faso
• Chile	• Colombia	• Bolivia	• Ethiopia
• Czech	• Dominican	• Cambodia	• Guinea
• Hungary	• Republic	• Cameroon	• Madagascar
• Netherlands	• Ecuador	• Ghana	• Malawi
• New Zealand	• Guatemala	• Haiti	• Mozambique
• Norway	• Guyana	• Honduras	• Rwanda
• Poland	• Jordan	• India	• Sierra Leone
• Puerto Rico	• Malaysia	• Indonesia	• The Gambia
• Spain	• Mexico	• Iran	• Uganda
• Sweden	• Namibia	• Kenya	
• United Kingdom	• Panama	• Morocco	
• United States	• Peru	• Myanmar	
• Uruguay	• Serbia	• Nepal	
	• South Africa	• Nicaragua	
	• Turkey	• Nigeria	
		• Pakistan	
		• Palestine	
		• Sri Lanka	
		• Tanzania	
		• Timor Leste	
		• Tunisia	
		• Zambia	

**Fig 3:**
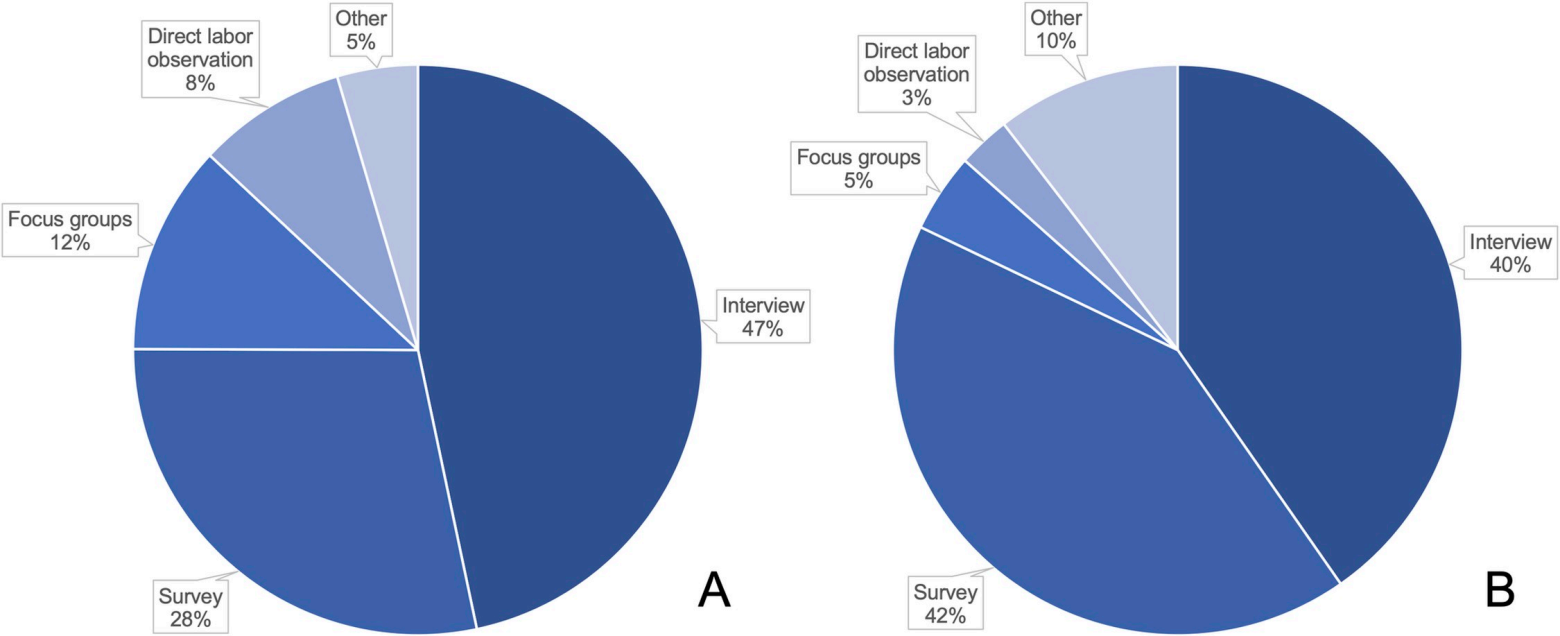
Distribution of research approaches used by (A) studies from all countries (B) studies in HICs.

The research approaches used in the 346 studies included quantitative methods (e.g., direct labor observation, surveys), qualitative methods (e.g., interviews, focus groups), and other approaches such as tool development and quality improvement projects (Fig 3A).

Out of the 346 included studies, only 61 were completed in HICs or in groups of countries containing at least one HIC. As some studies used more than one research approach, categorization of these studies into different research approaches yielded a total of 67 research approaches used in the 61 studies (Fig 3B) (Table 2).

**Table 2:** Studies in HICs categorized by research approach.

Research Approach	Studies	No. Studies
Survey	[13, 19–46]	29
Interview	[10, 41–44, 47–68]	27
Focus Groups	[47, 69, 70]	3
Direct Labor Observation	[66, 71]	2
Quality Improvement	[72, 73]	2
Other	[74–77]	4

Studies within each research approach were further examined for common themes of mistreatment adapted from those used by Bohren and colleagues [17]: neglect/abandonment, lack of privacy/confidentiality, lack of informed consent, verbal mistreatment, physical mistreatment, emotional mistreatment, health system constraints, and stigma and discrimination (Table 3).

**Table 3:** Themes of disrespectful care organized by research approach and contributing studies.

Type of Disrespectful Care	Research Approach	Studies	No. Studies	Total No. Studies
**Lack of Informed Consent** Overview: Studies reported mothers feeling as though they did not receive proper explanation or information about certain procedures and had no autonomy. Some women stated that examinations and procedures, including vaginal exams, cesarean sections, episiotomies, medications, injections, and stitches, were done against their will or without permission.	Survey	[13, 19, 22–24, 27–29, 33, 37–40, 42, 46]	15	34
	Interview	[42, 49, 50, 52, 54, 58, 59, 61, 64, 66, 68]	11	
	Focus Groups	[69, 70]	2	
	Direct Labor Observation	[66, 71]	2	
	Quality Improvement	[73]	1	
	Other	[74–76]	3	
**Stigma and Discrimination** Overview: Many women felt they received differential treatment and a lower quality of care based on a variety of factors. Some of the main factors were age, ethnicity, race, religion, number of previous births, disability status, education level, socioeconomic status, body weight, type of insurance, HIV status, and residential area.	Survey	[13, 19, 21, 23, 26, 28, 30, 32, 36, 37, 39–41, 45]	14	30
	Interview	[41, 47, 50, 53, 54, 56, 58, 59, 63, 67, 68]	12	
	Focus Groups	[47, 69]	2	
	Direct Labor Observation		0	
	Quality Improvement		0	
	Other	[74, 75]	2	
**Emotional Mistreatment** Overview: All forms of mistreatment can cause emotional distress, but emotional mistreatment was most commonly reported as women not being allowed to have a companion present during the birthing process ‐ including the father of the child.	Survey	[13, 19, 21, 22, 24–27, 29, 35, 39, 41, 46]	13	30
	Interview	[10, 41, 47–49, 54–56, 58, 61, 64, 67, 68]	13	
	Focus Groups	[47, 69, 70]	3	
	Direct Labor Observation		0	
	Quality Improvement	[73]	1	
	Other		0	
**Neglect/Abandonment** Overview: Many studies cited mothers being left alone for extended periods of time. In some studies, mothers were discouraged from having partners or family support in the labor room with them. Many mothers also faced long waiting times, whether in the waiting room itself, or waiting for procedures and labs to be completed.	Survey	[13, 19, 22, 24, 29, 35, 44, 46]	8	27
	Interview	[10, 44, 47, 49, 50, 52, 53, 55, 58–62, 65, 67]	15	
	Focus Groups	[69,70]	2	
	Focus Groups	[47, 69]	2	
	Direct Labor Observation		0	
	Quality Improvement		0	
	Other	[74, 75]	2	
**Mistreatment during Physical Exam Procedures** Overview: A range of mistreatment during PE procedures included unhygienic practices, performing procedures without offering or refusing use of anesthesia or analgesia, performing unindicated or painful vaginal exams, pushing hard on the abdomen, and performing procedures without consent.	Survey	[13, 19, 22, 25, 27, 31, 43]	7	24
	Interview	[10, 43, 49, 50, 54, 57, 58, 61, 62, 66, 68]	11	
	Focus Groups	[69]	1	
	Direct Labor Observation	[66, 71]	2	
	Quality Improvement		0	
	Other	[74–76]	3	
**Verbal Mistreatment** Overview: Verbal mistreatment encompassed scolding, mockery, humiliation, shouting, ordering to stop crying, insulting, blaming, and being threatened. This left many mothers who experienced this feeling ridiculed, stressed, and uncomfortable during the birthing process, adding to distrust for healthcare providers.	Survey	[13, 19, 21, 25, 27, 39, 40]	7	24
	Interview	[10, 49, 50, 52, 54, 56, 57, 60–62, 64, 67, 68]	13	
	Focus Groups	[69, 70]	2	
	Direct Labor Observation		0	
	Quality Improvement		0	
	Other	[74, 75]	2	
**Health Systems Constraints** Overview: Staffing shortages, quick staff turnover, lack of supplies, and provider burnout were identified as factors affecting quality of care.	Survey	[22, 26, 29, 32, 38, 44]	6	23
	Interview	[44, 48–50, 52, 54, 57–60, 62, 65, 66]	13	
	Focus Groups	[69]	1	
	Direct Labor Observation	[66]	1	
	Quality Improvement		0	
	Other	[74, 75]	2	
**Physical Mistreatment** Overview: Women who complained of physical mistreatment and the healthcare workers who observed it reported its manifestation in multiple forms. This includes pinching and slapping during the birthing process, as well as holding uncomfortable birthing positions or being restrained during birth.	Survey	[13, 19, 22, 25, 27]	5	15
	Interview	[49, 60, 61, 63, 64, 68]	6	
	Focus Groups	[70]	1	
	Direct Labor Observation		0	
	Quality Improvement		0	
	Other	[74–76]	3	
**Lack of Privacy/Confidentiality** Overview: Many mothers felt their health information was not protected and that healthcare providers did not respect patient-doctor confidentiality. Additionally, many felt that their privacy was not respected during labor, breast examinations, or vaginal examinations.	Survey	[13, 19, 22, 26, 38, 39]	6	15
	Interview	[54, 57, 61, 62, 67, 68]	6	
	Focus Group	[69]	1	
	Direct Labor Observation	[71]	1	
	Quality Improvement		0	
	Other	[75, 76]	1	

### Surveys

Twenty-nine studies included surveys of women regarding mistreatment during childbirth (Table 2). The most common forms of mistreatment reported by mothers included lack of informed consent (15/29), stigma and discrimination (14/29), and emotional mistreatment (13/29) (Table 3).

Studies addressing informed consent included themes such as method of labor, including vaginal and cesarean section, procedures during the labor process such as epidurals and inductions, and medical interventions such as a vaginal exam or episiotomy [13, 19, 22–24, 27–29, 33, 37–40, 42]. Specifically, many women felt as though they were coerced by medical professionals to make a decision quickly or to undergo a certain procedure or were not given enough information to make an informed choice [13, 22, 23, 28]. In the Giving Voice to Mother’s study by Vedam et al., one mother stated,

“The amount of times I felt coerced into decisions, or was mocked or rushed…,”

while another mother felt forced to have an episiotomy [13].

Stigma and discrimination came in several forms on the basis of race, ethnicity, sexual orientation, body weight, method of delivery, insurance type, and disability [13, 19, 21, 23, 26, 28, 30, 32, 36, 37, 39–41]. In many studies reporting stigma and discrimination during childbirth, women reported feeling discriminated against due to their race and/or ethnicity [13, 21, 23, 28, 36, 40]. Specifically, higher rates of mistreatment were reported for women of color, specifically Black, Latina, Asian, and Indigenous women [13, 36]. Women with government-funded health insurance reported being treated unfairly due to their race, ethnicity, or language spoken in comparison to women with private insurance [32]. Overall, themes arose for stigma and discrimination amongst women who somehow deviated from the norm of a “normal or desired” birth, such as those who had to receive a cesarean section or had a disability [26, 41].

Women reported emotional mistreatment surrounding lack of support or the forceful nature of healthcare providers [24, 39]. An account from a woman in a study by Murphy and Leah stated,

“…if someone comes with high voice and scolding you, you just keep quiet. You are just humiliated but you keep quiet” [39].

Two studies reported that women felt they had a negative birthing experience when they were not allowed to have a companion or support system with them in the room during the labor process [25, 41]. Emotional mistreatment once again arose as a theme in groups of women who were outside of the norm, such as those who had a lower education status or some form of disability [26, 35].

## Interviews

Twenty-seven studies utilized interviews to obtain personal commentaries on women’s birthing experiences (Table 2), allowing for qualitative analysis of maternal mistreatment based on the perceptions of the mothers themselves. The most common theme that mothers discussed during these interviews was neglect and abandonment (15/27) (Table 3) which was reported as feeling “forgotten about,” ignored, and lacking support [52, 55]. In one interview, a woman described being dismissed and left alone during labor, only to get help at the last minute when a midwife had barely enough time to put on one glove before catching the baby [10]. In another study, a woman reported that the healthcare workers at the institution she presented to persuaded her to return home, which resulted in her suffering a miscarriage with heavy bleeding in her bathroom [47].

The second most common theme reported by interviewed women was health system constraints (13/27) (Table 3). The most common complaint in this theme was a lack of continuity of care. Mothers reported “fragmented care” due to shift changes and staffing shortages, which hindered their ability to build a trusting relationship with their providers and feel comfortable during birth [49, 58]. Women also mentioned feeling frustrated that multiple different residents and physicians were seeing them and felt some were so busy that they did not have time to review the patient’s history prior to caring for them [65]. Mothers and healthcare workers described feeling constrained by hospital policies when they required a procedure to be done, since this limited provider and patient decision-making autonomy [66].

Other common themes that emerged during patient interviews were verbal and emotional mistreatment (13/27) (Table 3). Verbal mistreatment was often described as being screamed at, scolded, and degraded [49, 50, 52, 56]. As for emotional mistreatment, some mothers reported that nurses did not provide enough support and encouragement [24]. In one article, mothers were not allowed to have a support person in the room during the birthing process, which was associated with more negative perceptions of birth [41].

### Focus groups

Three studies conducted focus groups, with two focusing on patients’ perspectives [47, 69] and one focusing on doulas’ perspectives [70]. All three studies examined the outcomes and feelings toward the care provided to patients. People who performed the mistreatment included nurses, physicians, midwives, and other medical personnel.

Of the three studies, two reported neglect and abandonment [47, 69]. There were reports of loneliness and not being taken seriously [69] as well as accounts of women being persuaded to go home instead of receiving care and ending up experiencing miscarriages [47]. Two studies reported a lack of informed consent, specifically through communication and explanation in understandable terms of procedures being performed [69, 70]. Two studies reported verbal mistreatment with quotes from providers saying things such as describing the patient’s water breaking as “disgusting” [69] or making inappropriate jokes about “being in the wrong hole” and feeling disrespected [70]. One study reported physical mistreatment in the form of episiotomies being performed unexpectedly without pain medication, causing women to scream [47]. One study reported mistreatment during physical exams, with many different exams performed by different providers [69].

One of the three articles reported health system constraints, specifically discussing long wait times, inconsistency of care, and changing health care professionals due to changing shifts [69]. Two of the three articles additionally reported stigma and discrimination [47, 69]. There were reports of women of non-western backgrounds feeling like healthcare workers were becoming agitated with family members [69] as well as women feeling like they were treated with distrust and prejudice [47]. A common theme in all studies was that mistreatment and judgement against women was present due to their immigrant status [47], having lower incomes [69], or normalized behavior [70].

### Direct labor observations

Two studies utilized a direct labor observation approach to measure disrespectful care (Table 2). One of these studies took place in eight different countries, with only one country considered an HIC. In this study, seven midwives were observed during childbirth [71]. The other study took place in England and included the observation of 541 providers in the labor ward, almost half of whom were midwives or student midwives [66]. Both studies noted a lack of informed consent and mistreatment during physical exam procedures. Neither study noted neglect and abandonment, verbal, physical, emotional mistreatment, or stigma and discrimination (Table 3).

### Quality improvement

Two articles sought to evaluate the success of quality improvement initiatives (Table 2). The first quality improvement intervention was assigning one of four midwives to a woman for the duration of her pregnancy to build a trusting relationship and focus on treating the woman with dignity [72]. The intervention was considered successful, as women considered the midwives to promote a more positive birth experience. The second article assessed the impact of implementing a clinical guide for humanized care during delivery for three years [73]. The findings from this study indicated that the guidelines were poorly followed, and there was still a substantial percentage of women unsatisfied with their care during childbirth.

### Other research approaches

The remaining four studies employed diverse research approaches, including analyzing social media content [75], using descriptive phenomenology [76], performing qualitative secondary analysis [74], and developing a questionnaire [77]. The most common themes of mistreatment in these studies were mistreatment during physical exam procedures, lack of informed consent, and physical mistreatment (Table 2). The study analyzing social media content found 50 stories involving physical mistreatment. One woman described being

"…involuntarily catheterized during birth. I yelled NO. According to the nurse, this is ‘how it is done’. Two people were holding down my legs." [75].

## Discussion

In this scoping review, we examined current qualitative and quantitative RMC research depending on research approach, study location, and country income. This provides important information for researchers regarding which research approaches are being used in certain locations and how these different research approaches assess disrespectful maternity care in healthcare facilities. The articles included in this review show that while RMC research has progressed and increased over the past two decades, current research frames disrespectful care as an issue for primarily L/LMICs instead of a global health concern. This is the first attempt to summarize RMC research by research approach, to analyze the various approaches by their ability to identify mistreatment during childbirth specifically in HICs, and to identify gaps in research in locations throughout the world.

The multiple research approaches analyzed in this review offer different insight into maternal mistreatment. Direct labor observations, for example, offer objective analysis of care during childbirth as labor is progressing [66, 71], while surveys collect patient experiences afterward [13, 19–46]. Patient and provider interviews provide direct patient insight and shed light on the emotional trauma of disrespectful care [10, 41–44, 47–68]. Focus groups allow for the synthesis of common themes among women with various birthing experiences [47, 69, 70]. For studies taking place in HICs, surveys [13, 19–46], interviews [10, 41–44, 47, 68], and focus groups [47, 69, 70] identified all nine types of mistreatment analyzed in this review. Among these three research approaches, lack of informed consent, emotional mistreatment, and stigma/discrimination were most frequently reported.

Interestingly, certain research approaches were utilized more in certain country income classes. For example, direct labor observations made up only 3% of the research approaches utilized in HICs versus 9% in all articles included in this review (Fig 2). Considering the sensitive nature of entering into a mother’s room during labor and observing for a long period of time, direct labor observations may be harder to conduct in HICs due to more stringent research and ethical regulations [78]. If this research approach is considered more carefully in HICs, then the ethical implications of conducting direct labor observations in vulnerable populations in L/LMICs should be considered.

Organizing the studies by geographical location identified many countries that lack RMC research (Fig 2). Six countries (Tanzania, Nigeria, Kenya, India, Brazil, and Ethiopia) represented over 40% of research analyzed out of the total 66 countries represented. The WHO’s estimates from 2017 indicate that 86% of maternal deaths took place in Sub-Saharan Africa and South Asia. In particular, Nigeria and India, which have high birth rates, accounted for 35% of worldwide maternal deaths [79]. Thus, researching and analyzing RMC in these vulnerable populations continues to be important for improving maternal wellbeing and lowering the MMR.

Thus far, RMC research has largely focused on L/LMICs. In 2017, the WHO estimated the MMR in LICs to be about 40 times higher than that in Europe and 60 times higher than that in Australia and New Zealand [79]. These statistics indicate the importance of continuing research that focuses on topics such as RMD that could potentially improve maternal health outcomes in L/LMICs. However, this review and recent research identifies a need to conduct research in all global settings. Out of the 346 studies included in this review, 61 studies (17.6%) were conducted in HICs. All nine types of mistreatment analyzed in this review, including physical mistreatment, were identified in studies taking place in HICs. However, HICs have declared their commitment to reducing their MMRs. The US Office of Disease Prevention and Promotion included reducing maternal mortality in their 2030 Healthy People Objectives. Likewise, the United Kingdom’s government aims to reduce MMR disparities amongst Black women and cut their MMR in half by 2025 [80, 81]. Further research is needed in HICs, particularly related to maternity care for those that are marginalized, including Black and native women [82]. The paucity of research in HICs may reinforce biases that lack of respectful maternity care is a problem only in other countries. While giving birth in a well-equipped facility with well-trained healthcare providers reduces maternal morbidity and mortality, lack of respectful care in any country contributes to poor outcomes [3–12]. With the increasing MMR seen in the United States and increasing MMR disparities, particularly among marginalized groups, it is crucial to explore RMC in all countries working to improve maternal health outcomes.

There are some limitations to this scoping review. Due to the broad range of terminology used in the RMC field of research, it is possible that some relevant articles were not retrieved by our database searches. The quality of articles was not critically appraised; thus, the conclusions of this review are based on the existence of studies rather than their quality. This review focuses on articles indexed in PubMed/MEDLINE, EMBASE, CINAHL Complete, and the Maternity & Infant Care Database and possibly excluded relevant studies that were indexed in other databases or communicated as grey literature.

## Conclusion

While RMC research has expanded over the past two decades, there is a gap in research focusing on HICs. Various research approaches used to measure RMC in HICs identify the presence of all nine types of mistreatment analyzed in this review, including physical mistreatment. The lack of research in HICs may lead to biases that respectful maternity care is not an issue in these countries; however, this review provides strong evidence that disrespectful care also exists in HICs. Lack of respectful maternity care in any country contributes to poor outcomes, particularly in marginalized groups [3–12, 82]. More research in HICs is needed to provide a better picture of RMC and lead to the development of interventions that embrace an equitable, patient-centric empowerment model of maternity care and improve outcomes in childbearing patients.

## Supporting information

S1 File Preferred reporting items for systematic reviews and meta-analyses extension for scoping reviews [PRISMA-ScR] checklist. (PDF)

S2 File Search terms utilized for initial article search. (DOCX)

S1 Table Characteristics of analyzed HIC articles. (XLSX)

S2 Table Data charting for analyzed HIC articles. (XLSX)

S3 Table Reference list of all included articles. (XLSX)
