# Supplementary material for: Approaches and geographical locations of respectful maternity care research: A scoping review
Source: PLoS One. 2023 Aug 24;18(8):e0290434. doi: 10.1371/journal.pone.0290434 (PMC10449213; doi:10.1371/journal.pone.0290434)
Supplement: S2 File — (DOCX) [file pone.0290434.s002.docx]

**Complete Search Strategies**

***PubMed/MEDLINE***

(("delivery, obstetric"[Mesh] OR "perinatal care"[Mesh] OR “perinatal care”[tiab] OR “perinatal healthcare”[tiab] OR “perinatal health care”[tiab] OR “perinatal service”[tiab] OR “perinatal services”[tiab] OR “perinatal health service”[tiab] OR “perinatal health services”[tiab] OR “maternal health services"[Mesh] OR “maternal care”[tiab] OR “maternal healthcare”[tiab] OR “maternal health care”[tiab] OR “maternal service”[tiab] OR “maternal services”[tiab] OR “maternal health service”[tiab] OR “maternal health services”[tiab] OR “maternity care”[tiab] OR “maternity healthcare”[tiab] OR “maternity health care”[tiab] OR “maternity service”[tiab] OR “maternity services”[tiab] OR “maternity health service”[tiab] OR “maternity health services”[tiab]) AND (birth[tiab] OR births[tiab] OR childbirth[tiab] OR childbirths[tiab] OR delivery[tiab] OR deliveries[tiab])) AND (“disrespect”[tiab] OR “disrespects”[tiab] OR “disrespectful”[tiab] OR “disrespected”[tiab] OR “respectful”[tiab] OR “abuse”[tiab] OR “abused”[tiab] OR “abusive”[tiab] OR “abuses”[tiab] OR “neglect”[tiab] OR “neglected”[tiab] OR “neglects”[tiab] OR “neglectful”[tiab] OR “confidentiality”[tiab] OR “confidential”[tiab] OR “non-confidential”[tiab] OR “informed consent”[tiab] OR “violence”[tiab] OR “violent”[tiab] OR “humiliation”[tiab] OR “humiliate”[tiab] OR “humiliating”[tiab] OR “condescend”[tiab] OR “condescending”[tiab] OR “condescension”[tiab] OR “intimidation”[tiab] OR “intimidate”[tiab] OR “intimidating”[tiab] OR “yelling”[tiab] OR “yell”[tiab] OR “non dignified”[tiab] OR “non-dignified”[tiab] OR “undignified”[tiab] OR “discrimination”[tiab] OR “discriminate”[tiab] OR “discriminated”[tiab] OR “abandon”[tiab] OR “abandoned”[tiab] OR “abandonment”[tiab] OR “detain”[tiab] OR “detained”[tiab] OR “detention”[tiab] OR “human rights”[tiab] OR “maltreatment”[tiab] OR “maltreated”[tiab] OR “mistreatment”[tiab] OR “mistreated”[tiab] OR “dehumanized”[tiab] OR “dehumanization”[tiab] OR “dignity”[tiab] OR “dignified”[tiab] OR “undignified”[tiab] OR “stigma”[tiab] OR “bullying”[tiab] OR “bully”[tiab]) NOT ("animals"[Mesh] NOT "humans"[Mesh])

**EMBASE**

((“obstetric delivery”/exp OR "perinatal care"/exp OR “perinatal care”:ti,ab OR “perinatal healthcare”:ti,ab OR “perinatal health care”:ti,ab OR “perinatal service”:ti,ab OR “perinatal services”:ti,ab OR “perinatal health service”:ti,ab OR “perinatal health services”:ti,ab OR “maternal care”:ti,ab OR “maternal healthcare”:ti,ab OR “maternal health care”:ti,ab OR “maternal health service"/exp OR “maternal service”:ti,ab OR “maternal health service”:ti,ab OR “maternal services”:ti,ab OR “maternal health services”:ti,ab OR “maternity care”:ti,ab OR “maternity healthcare”:ti,ab OR “maternity health care”:ti,ab OR “maternity service”:ti,ab OR “maternity health service”:ti,ab OR “maternity services”:ti,ab OR “maternity health services”:ti,ab) AND (birth:ti,ab OR births:ti,ab OR childbirth:ti,ab OR childbirths:ti,ab OR delivery:ti,ab OR deliveries:ti,ab)) AND (“disrespect”:ti,ab OR “disrespects”:ti,ab OR “disrespectful”:ti,ab OR “disrespected”:ti,ab OR “respectful”:ti,ab OR “abuse”:ti,ab OR “abused”:ti,ab OR “abusive”:ti,ab OR “abuses”:ti,ab OR “neglect”:ti,ab OR “neglected”:ti,ab OR “neglects”:ti,ab OR “neglectful”:ti,ab OR “confidentiality”:ti,ab OR “confidential”:ti,ab OR “non-confidential”:ti,ab OR “informed consent”:ti,ab OR “violence”:ti,ab OR “violent”:ti,ab OR “humiliation”:ti,ab OR “humiliate”:ti,ab OR “humiliating”:ti,ab OR “condescend”:ti,ab OR “condescending”:ti,ab OR “condescension”:ti,ab OR “intimidation”:ti,ab OR “intimidate”:ti,ab OR “intimidating”:ti,ab OR “yelling”:ti,ab OR “yell”:ti,ab OR “non dignified”:ti,ab OR “non-dignified”:ti,ab OR “undignified”:ti,ab OR “discrimination”:ti,ab OR “discriminate”:ti,ab OR “discriminated”:ti,ab OR “abandon”:ti,ab OR “abandoned”:ti,ab OR “abandonment”:ti,ab OR “detain”:ti,ab OR “detained”:ti,ab OR “detention”:ti,ab OR “human rights”:ti,ab OR “maltreatment”:ti,ab OR “maltreated”:ti,ab OR “mistreatment”:ti,ab OR “mistreated”:ti,ab OR “dehumanized”:ti,ab OR “dehumanization”:ti,ab OR “dignity”:ti,ab OR “dignified”:ti,ab OR “undignified”:ti,ab OR “stigma”:ti,ab OR “bullying”:ti,ab OR “bully”:ti,ab) NOT ("animals"/exp NOT "humans"/exp)

***CINAHL Complete***

((MH "Maternal-Child Care+" OR MH “Obstetric Patients+” OR MH “Rooming In+” OR MH “Obstetric Service+” OR MH “Childbirth+” OR MH "Women's Health Services/EI" OR MH "Obstetric Care" OR TI “perinatal care” OR TI “perinatal healthcare” OR TI “perinatal health care” OR TI “perinatal service” OR TI “perinatal services” OR “perinatal health service” OR “perinatal health services” OR TI “maternal care” OR TI “maternal healthcare” OR TI “maternal health care” OR TI “maternal service” OR TI “maternal services” OR TI “maternal health service” OR TI “maternal health services” OR TI “maternity care” OR TI “maternity healthcare” OR TI “maternity health care” OR TI “maternity service” OR TI “maternity services” OR TI “maternity health service” OR TI “maternity health services” OR AB “perinatal care” OR AB “perinatal healthcare” OR AB “perinatal health care” OR AB “perinatal service” OR AB “perinatal services” OR AB “perinatal health service” OR AB “perinatal health services” OR AB “maternal care” OR AB “maternal healthcare” OR AB “maternal health care” OR AB “maternal service” OR AB “maternal services” OR AB “maternal health service” OR AB “maternal health services” OR AB “maternity care” OR AB “maternity healthcare” OR AB “maternity health care” OR AB “maternity service” OR AB “maternity services” OR AB “maternity health service” OR AB “maternity health services”) AND (TI birth OR TI births OR TI childbirth OR TI childbirths OR TI delivery OR TI deliveries OR AB birth OR AB births OR AB childbirth OR AB childbirths OR AB delivery OR AB deliveries)) AND (TI “disrespect” OR TI “disrespects” OR TI “disrespectful” OR TI “disrespected” OR TI “respectful” OR TI “abuse” OR TI “abused” OR TI “abusive” OR TI “abuses” OR TI “neglect” OR TI “neglected” OR TI “neglects” OR TI “neglectful” OR TI “confidentiality” OR TI “confidential” OR TI “non-confidential” OR TI “informed consent” OR TI “violence” OR TI “violent” OR TI “humiliation” OR TI “humiliate” OR TI “humiliating” OR TI “condescend” OR TI “condescending” OR TI “condescension” OR TI “intimidation” OR TI “intimidate” OR TI “intimidating” OR TI “yelling” OR TI “yell” OR TI “non dignified” OR TI “non-dignified” OR TI “undignified” OR TI “discrimination” OR TI “discriminate” OR TI “discriminated” OR TI “abandon” OR TI “abandoned” OR TI “abandonment” OR TI “detain” OR TI “detained” OR TI “detention” OR TI “human rights” OR TI “maltreatment” OR TI “maltreated” OR TI “mistreatment” OR TI “mistreated” OR TI “dehumanized” OR TI “dehumanization” OR TI “dignity” OR TI “dignified” OR TI “undignified” OR TI “stigma” OR TI “bullying” OR TI “bully” OR AB “disrespect” OR AB “disrespects” OR AB “disrespectful” OR AB “disrespected” OR AB “respectful” OR AB “abuse” OR AB “abused” OR AB “abusive” OR AB “abuses” OR AB “neglect” OR AB “neglected” OR AB “neglects” OR AB “neglectful” OR AB “confidentiality” OR AB “confidential” OR AB “non-confidential” OR AB “informed consent” OR AB “violence” OR AB “violent” OR AB “humiliation” OR AB “humiliate” OR AB “humiliating” OR AB “condescend” OR AB “condescending” OR AB “condescension” OR AB “intimidation” OR AB “intimidate” OR AB “intimidating” OR AB “yelling” OR AB “yell” OR AB “non dignified” OR AB “non-dignified” OR AB “undignified” OR AB “discrimination” OR AB “discriminate” OR AB “discriminated” OR AB “abandon” OR AB “abandoned” OR AB “abandonment” OR AB “detain” OR AB “detained” OR AB “detention” OR AB “human rights” OR AB “maltreatment” OR AB “maltreated” OR AB “mistreatment” OR AB “mistreated” OR AB “dehumanized” OR AB “dehumanization” OR AB “dignity” OR AB “dignified” OR AB “undignified” OR AB “stigma” OR AB “bullying” OR AB “bully”)

***Maternity & Infant Care Database***

Title, abstract, or heading word: ((perinatal OR maternal OR maternity) AND (healthcare OR health care OR service OR services OR health service OR health services) AND (birth OR births OR childbirth OR childbirths OR delivery OR deliveries)) AND (disrespect OR disrespects OR disrespectful OR disrespected OR respectful OR abuse OR abused OR abusive OR abuses OR neglect OR neglected OR neglects OR neglectful OR confidentiality OR confidential OR non-confidential OR informed consent OR violence OR violent OR humiliation OR humiliate OR humiliating OR condescend OR condescending OR condescension OR intimidation OR intimidate OR intimidating OR yelling OR yell OR non dignified OR non-dignified OR undignified OR discrimination OR discriminate OR discriminated OR abandon OR abandoned OR abandonment OR detain OR detained OR detention OR human rights OR maltreatment OR maltreated OR mistreatment OR mistreated OR dehumanized OR dehumanization OR dignity OR dignified OR undignified OR stigma OR bullying OR bully)
